# Chemical and microbial characterization of sugarcane mill mud for soil applications

**DOI:** 10.1371/journal.pone.0272013

**Published:** 2022-08-16

**Authors:** Minori Uchimiya, Anthony G. Hay, Jeffrey LeBlanc

**Affiliations:** 1 Southern Regional Research Center, Agricultural Research Service, United States Department of Agriculture, New Orleans, Louisiana, United States of America; 2 Department of Microbiology, Cornell University, Ithaca, New York, United States of America; 3 Higginbotham Farm, Washington, Louisiana, United States of America; Gifu University, JAPAN

## Abstract

Sugarcane mill mud/filter cake is an activated sludge-like byproduct from the clarifier of a raw sugar production factory, where cane juice is heated to ≈90°C for 1–2 hr, after the removal of bagasse. Mill mud is enriched with organic carbon, nitrogen, and nutrient minerals; no prior report utilized 16S rRNA gene sequencing to characterize the microbial composition. Mill mud could be applied to agricultural fields as biofertilizer to replace or supplement chemical fertilizers, and as bio-stimulant to replenish microorganisms and organic carbon depleted by erosion and post-harvest field burning. However, mill mud has historically caused waste management challenges in the United States. This study reports on the chemical and microbial (16S rRNA) characteristics for mill muds of diverse origin and ages. Chemical signature (high phosphorus) distinguished mill mud from bagasse (high carbon to nitrogen (C/N) ratio) and soil (high pH) samples of diverse geographical/environmental origins. Bacterial alpha diversity of all sample types (mill mud, bagasse, and soil) was inversely correlated with C/N. Firmicutes dominated the microbial composition of fresh byproducts (mill mud and bagasse) as-produced within the operating factory. Upon aging and environmental exposure, the microbial community of the byproducts diversified to resemble that of soils, and became dominated by varying proportions of other phyla such as Acidobacteria, Chloroflexi, and Planctomyces. In summary, chemical properties allowed grouping of sample types (mill mud, bagasse, and soil-like), and microbial diversity analyses visualized aging caused by outdoor exposures including soil amendment and composting. Results suggest that a transient turnover of microbiome by amendments shifts towards more resilient population governed by the chemistry of bulk soil.

## Introduction

Land application of crop residues from sugar processing and other agricultural/food commodity production provides a promising path towards sustainable waste management [1]. Bagasse (fibrous residue after extracting juice from sugarcane stalk) is traditionally used to fuel boilers at sugarcane mill/factory to produce raw sugar from cane juice [2]. Numerous other potential uses of bagasse have been proposed, including direct and indirect (e.g., after composting) soil application. However, end-user adaptation is currently limited, partly because bagasse is bulky and has low nutrient density compared to mill mud [3]. Mill mud contains all solid impurities from converting sugarcane juice to raw sugar under elevated temperature, including residual soil, phosphate precipitates, calcium oxalate/aconitate, sucrose, fiber, and protein [1]. Consequently, mill mud is a promising organic fertilizer enriched with organic carbon, nitrogen and other essential plant nutrients [3]. In Florida, mill mud (without composting, mulching, or other pre-treatments) is applied to improve sugarcane yields [4]. Mill mud is used to restore the fertility of sandy soils (low organic carbon) to the level of organic “muck” soil (Histosols), which are becoming depleted because of microbial oxidation of soil organic carbon [5]. Similar organic carbon depletion in Louisiana sugarcane fields is caused by erosion [6] and by biomass burning [7]. There are several advantages of sugarcane mill mud over other nutrient-dense materials for soil conservation. For example, mill mud could contain plant nutrients equivalent to manure [8]; yet, mill mud is partially sterilized by the elevated temperature treatments during sugar processing [2]. Fly ash (byproduct of bagasse burning to generate energy during raw sugar production) is applied to sugarcane fields to supplement mineral nutrients, but lacks organic carbon and microbiome. In summary, mill mud is uniquely enriched with nutrients, organic carbon, and microbial biomass [9] to satisfy both biofertilizer and bio-stimulant [10] applications.

A few recent studies emerged to investigate mill mud’s effects on microbial carbon use efficiency [11], soil compaction [12], and aggregate stability [13]. However, environmental fate of mill mud’s native microbial community and its driver are largely unknown. To our knowledge, no prior report utilized 16S rRNA gene sequencing to identify microbial composition of sugarcane mill mud before and after environmental exposure. Nutrients and other chemical composition is expected to drive community-level functions of soil amendments [14]. The objective of this study was to characterize sugarcane mill mud of diverse origins, with the ultimate goal of understanding its impact on soil after field application. We hypothesized that chemistry of mill mud controls stable taxonomic composition, because phylogeny and function are strongly correlated [15]. To test this hypothesis, field samples were collected to represent varying degrees of aging by environmental exposure. Mill mud and bagasse samples collected indoor within the operating factory will serve as the baseline without field aging. Mill mud and bagasse stored outdoor for several months represent intermediate levels of aging. Finally, soils (silt loams) serve as the longest outdoor exposure endpoint, as opposed to bagasse and mill mud collected indoor within the factory.

## Materials and methods

### Field samples

A total of 15 mill mud, bagasse, and soil samples were collected from five different sugarcane factories in Louisiana (LA1, LA2, and LA3) and Florida (FL1 and FL2): LA1 (soil, factory mud, legacy mud, and factory bagasse) and LA2 (soil, factory mud, and factory bagasse) during the 2019 harvest season (October-December, 2019); LA3 (aged bagasse, legacy mud, aged mud, amended soil, and soil) prior to the 2019 harvest season (July, 2019); and FL1 (factory mud) and FL2 (factory mud and legacy mud) during the 2019 harvest season. Abbreviations for 15 samples are provided in Table 1, and observations from field sampling are described below. During the 2019 harvest season, factory mud (Mud-LA1, Mud-LA2, Mud-FL1, and Mud-FL2 in Table 1) was collected directly from the conveyor belt press filter within the operating sugarcane processing factory; factory bagasse (Bagasse-LA1 and Bagasse-LA2 in Table 1) was collected from the conveyor belt immediately following the cane crushers. Therefore, a total of 6 factory mud and factory bagasse samples in Table 1 (Mud-LA1, Mud-LA2, Mud-FL1, Mud-FL2, Bagasse-LA1, and Bagasse-LA2) were collected indoors, within the operating sugarcane mills. All other samples in Table 1 were collected outside of the factory, and were exposed to varying degrees of environmental exposure, as described below.

**Table 1:** Abbreviations for 15 mill mud, bagasse, and soil samples. Different text styles are used to highlight the degree of sample aging: mill mud and bagasse without aging (collected indoor within the operating sugarcane factory, plain text), samples aged by varying degrees of environmental exposure (italicized), and soil without mill mud or bagasse for comparison (underlined).

location	factory	*legacy*	*aged*	factory	*aged*	soil	*amended*
	mud	*mud*	*mud*	bagasse	*bagasse*		*soil*
**LA1**	Mud-LA1	*Mud-LA1-O*	* *	Bagasse-LA1	* *	Soil-LA1	
**LA2**	Mud-LA2		* *	Bagasse-LA2	* *	Soil-LA2	
**LA3**		*Mud-LA3-O*	*Mud-LA3*		*Bagasse-LA3*	Soil-LA3	*Mud-LA3-S*
**FL1**	Mud-FL1		* *				
**FL2**	Mud-FL2	*M-FL2-P*	* *				

All samples from LA3 location were collected outdoors. At LA3 location, aged bagasse and aged mud were piled outdoors for 9–10 months with (Bagasse-LA3 to keep bagasse dry) or without (Mud-LA3) roofing. Those samples represent byproducts that were temporarily stored outdoor for utilization in the subsequent harvest season to fuel the boiler (Bagasse-LA3) or to apply on sugarcane fields (Mud-LA3). This aged mud (Mud-LA3) was applied to a sugarcane field in spring 2019 (before planting) using a manure spreader. In summer 2019 (after planting), top soil (0–10 cm) [16] from this site was collected as amended soil (Mud-LA3-S in Table 1). For comparison, another soil sample was collected from an adjacent field before mill mud application and sugarcane planting took place (Soil-LA3 in Table 1). Although mill mud was applied to this site in previous year, Soil-LA3 was visibly sandy and had less organic matter compared to Mud-LA3-S. Legacy mud (Mud-LA3-O in Table 1) was collected from a naturally composting dry pile with high sand content, which had historically been used as a disposal site for mill mud, ash, and bagasse generated on-site.

Legacy mud from LA1 location (mud-LA1-O) was a dense slurry from an on-site waste pond, and was composed primarily of sugarcane mill mud. Soil samples from LA1 and LA2 had no known history of mill mud application (soil-LA1 and soil-LA2 in Table 1), and were collected using a gardening spade (0–10 cm).

### Sample analysis

Chemical characterization was conducted by the Cornell Nutrient Analysis Laboratory (Test 3020; Ithaca, NY) following standardized methods for compost [17]. Briefly, pH (1:5 = solid:water) and soluble salts (by conductivity) were measured using dedicated electrodes. Ammonium and nitrate contents were measured by colorimetric analysis of KCl extracts. Combustion method was used for % solid, % organic matter, and ash contents; gas chromatography was used to measure carbon and nitrogen contents to calculate carbon to nitrogen ratio (C/N). Phosphorus (P), potassium (K), calcium (Ca), magnesium (Mg), sodium (Na), iron (Fe), copper (Cu), manganese (Mn), and aluminum (Al) contents were measured by acid digestion and inductively coupled plasma optical emission spectrometer (ICP-OES). 16S rRNA gene sequencing was conducted by the Cornell Sequencing Center (Ithaca, NY), as described in detail below.

### DNA extraction

Frozen samples were thawed on ice, mixed for 30 s using a sterile hand-held metal spatula, and then 0.25 g wet weight was placed in 2 mL tubes with 0.25 mL of 0.1 mm glass beads [18]. Lysis solution (1 mL) from E.Z.N.A.® Soil DNA Kit (OMEGA Bio-Tek, Doraville, USA) was added to the tube, which was then capped and beadbeated on a Biospec tissue homogenizer for 1 min. After centrifugation at 15000 x g, 250 μL of supernatant was processed according to the manufacturer’s instructions. Bagasse-LA3 and Soil-LA1 (Table 1) were selected for triplicate extraction as process controls, because they had a more heterogeneous appearance.

### Sequencing

The extracted DNA (1 ng per sample) was subjected to PCR amplification targeting the V4 region of the 16S rRNA gene using barcoded primers (515F 5’-CCTACGGGAGGCAGCAG-3’ and 806R 5’-GGACTACHVGGGTWTCTAAT-3’) using high-fidelity polymerase (New England BioLabs, Ipswich, MA). The V4 is widely used in microbial ecology studies and therefore allows for direct comparisons in the future with 16S communities from other locations [19, 20]. The PCR products were purified and normalized on a Sequalprep plate (Applied Biosystems, Waltham MA), and then pooled. Ten pg of phiX was added as a control. Paired-end sequencing (2 × 250 bp) was carried out on an Illumina MiSeq sequencer at the Cornell Sequencing Center.

### Sequence processing

Raw sequences were uploaded to QIITA and processed through the standard workflow [21]. Briefly, they were trimmed, chloroplasts and mitochondrial sequences were filtered as were all singletons. The resultant Deblur table was used as the basis for Alpha diversity (Shannon, Chao 1, Faith’s Phylogenetic Diversity) and Beta diversity analysis. The deblur table condensed to the phylum level was exported as a csv file and used as the basis for Beta diversity analyses via principle component (PCA) and heatmap analyses in Clust-Vis [22].

### Statistical analyses

The chemical data were initially evaluated for correlations in Excel to eliminate redundant values derived from the same data and to reduce the number of features analyzed for the PCA. For example, when two variables were tightly correlated and were methodologically derived from one or more shared empirical measurements, only one was included. Both the PCA and the heatmap were generated in Clustvis [23] via implementation of the pcaMethods and pheatmap packages in R using default settings. The PCAs were calculated using Single Value Decomposition with imputation. The ellipses on the PCAs delineate the 95% confidence intervals in which a new observation from the same group (indicated by color coding) would likely be found. The heatmap rows were scaled to unit variance (within row values were divided by standard deviation so that each row has variance equal to one). Cladograms above each heat map are graphical representations of quantitative similarity between samples as calculated using complete linkage clustering: the shorter the vertical line between samples on the same node (sharing a horizontal bar), the more similar they are. Groupings based on physicochemical properties were then used to interpret PCA and cluster analyses of 16S rRNA data. Between group/clade statistical comparisons of individual measures were determined via analysis of variance (ANOVA) followed by Tukey’s honestly significant difference (HSD) using iCalcu [24]. Sample types were considered significantly different from one another when the P <0.05.

## Results

### Grouping of samples by chemical properties

**Fig 1:**
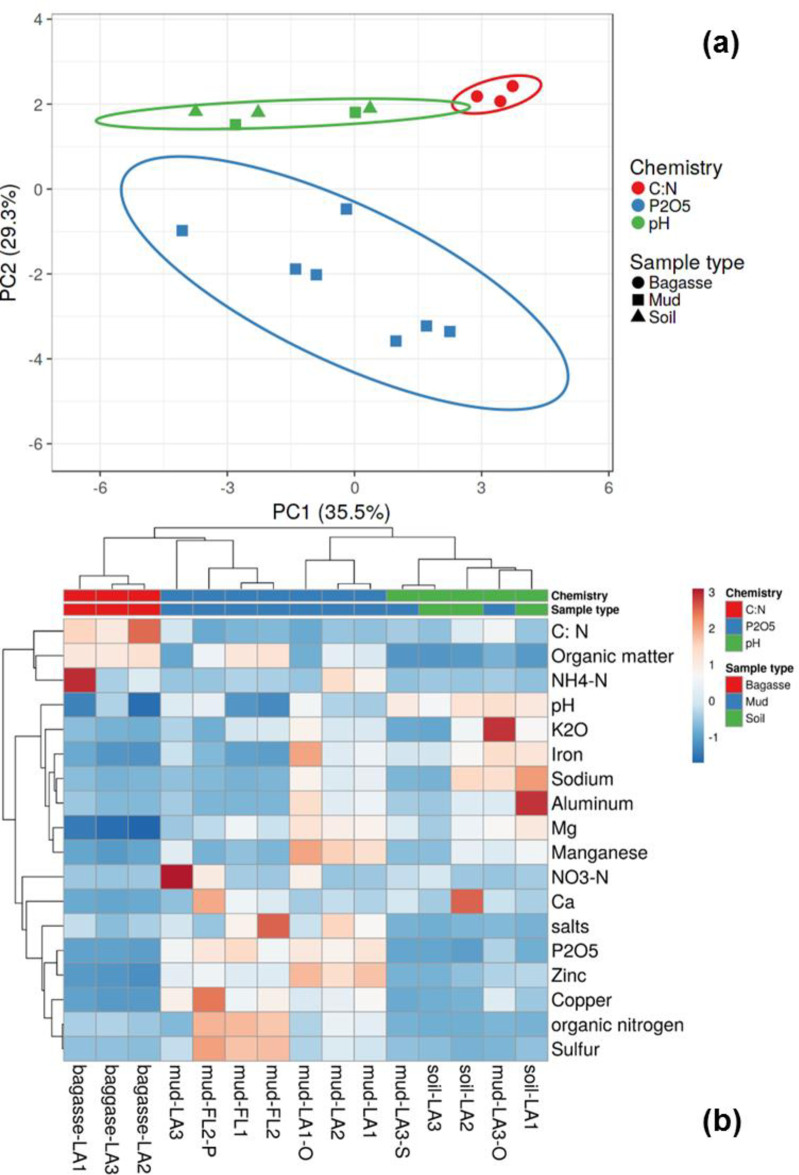
Chemical analysis of bagasse, soil, and mud collected from sugarcane production facilities. (a) Principal components analysis and (b) heatmap with complete linkage clustering. Results were normalized to within row variance with the colored scale bar representing standard deviations from within-row means [23].

Principal component (PC) analysis of the chemical composition grouped samples into 3 main clusters (Fig 1A). PC1 and PC2 explained 36.9% and 29.3% of the variance, respectively. The ellipses around the symbols indicate the 95% confidence intervals of classes: bagasse, mud, and soil. The chemical class labels for the three clusters (C:N, P_2_O_5_, pH) were based on the statistical significance of each measurement based on sample type. The bagasse samples (circles) grouped together and were notable for high C:N average ratio (>97, colored red in Fig 1A). Most of the mud samples (squares) grouped together and averaged >2 mg/kg P_2_O_5_ (colored blue) (Table 2). As described in Materials and Methods, legacy mud from LA3 location (Mud-LA3-O in Table 1) had high sand content, and grouped together with LA3 soil amended with aged mud (Mud-LA3-S) as well as soils from other locations (triangles representing Soil-LA1, Soil-LA2, and Soil-LA3) having pH >7.3 (colored green in Fig 1A).

**Table 2:** Average values of chemistry clades by sample type (^†^based on heatmap clustering in Fig 1, mud-LA3-S and mud-LA3-O were included as soils due to their chemical similarity to other soils). Within-column values with the different super script letters were significantly different from one another as determined by Tukey’s HSD: pH of bagasse vs. soil = 2.7553 (p = 0.0009); mud vs. soil = 1.722 (p = 0.0057). P_2_O_5_ of bagasse vs. mud 2.0476 (p = 0); mud vs. soil = 1.8543 (p = 0); C:N of bagasse vs. mud = 74.469 (p = 0); bagasse vs. soil = 60.3313 (p = 0.0003).

Distinguishing Feature	pH	P_2_O_5_	C:N
Bagasse (n = 3)	5.19±0.85^A^	0.13±0.01^C^	97.5±22.5^E^
Mud (n = 7)	6.93±0.9^A^	1.4±(0.34)^D^	28.9±9.23^F^
Soil (n = 5)^†^	7.94±0.36^B^	0.32±0.28^C^	37.2±16.3^F^

In the heatmap detailing chemical similarity (Fig 1B), each block is colored to represent the number of standard deviations from the mean based on within-row variance. Thus, within each row, higher than average samples are colored red and lower than average samples are colored blue. Color bars above the heatmap show sample membership in chemically distinct groups (row 1: C:N, P_2_O_5_, pH) or the sample type (row2: bagasse, mud, soil). Overall, there was good alignment between chemistry (row 1) and labels based on observations from field sampling (row 2): C/N for bagasse (red), P_2_O_5_ for mill mud (blue) and pH for soil (green). As described above, soil amended with mill mud (Mud-LA3-S) and legacy mud from LA3 (Mud-LA3-O) were chemically classified as “soil” in Fig 1B, because of high mineral contents.

Overall, Fig 1B illustrates signature chemical parameters for mud (red clusters in center bottom), soil (red clusters in middle right), and bagasse (small red cluster in upper left corner). Signature chemical parameter for mud (P_2_O_5_) clustered with other mineral nutrients (e.g., Cu, S). For soil group, pH clustered with alkali and alkaline earth metals. Bagasse was represented by high C/N and organic matter. In summary, Fig 1 provided a fingerprint of chemical parameters for classifying field samples to mill mud, soil, and bagasse categories. Samples with high mineral contents (e.g., Mud-LA3-S and Mud-LA3-O) were therefore chemically classified as soil in Fig 1, although those samples contained some mill mud. In subsequent sections of this work focusing on the microbiome, the statistically significant differences between sample type chemistry (Table 2) was used to determine correlations with community membership and structure: bagasse (high C/N), mud (high P_2_O_5_), soil (high pH).

### Microbial 16S rRNA gene community analyses

**Fig 2:**
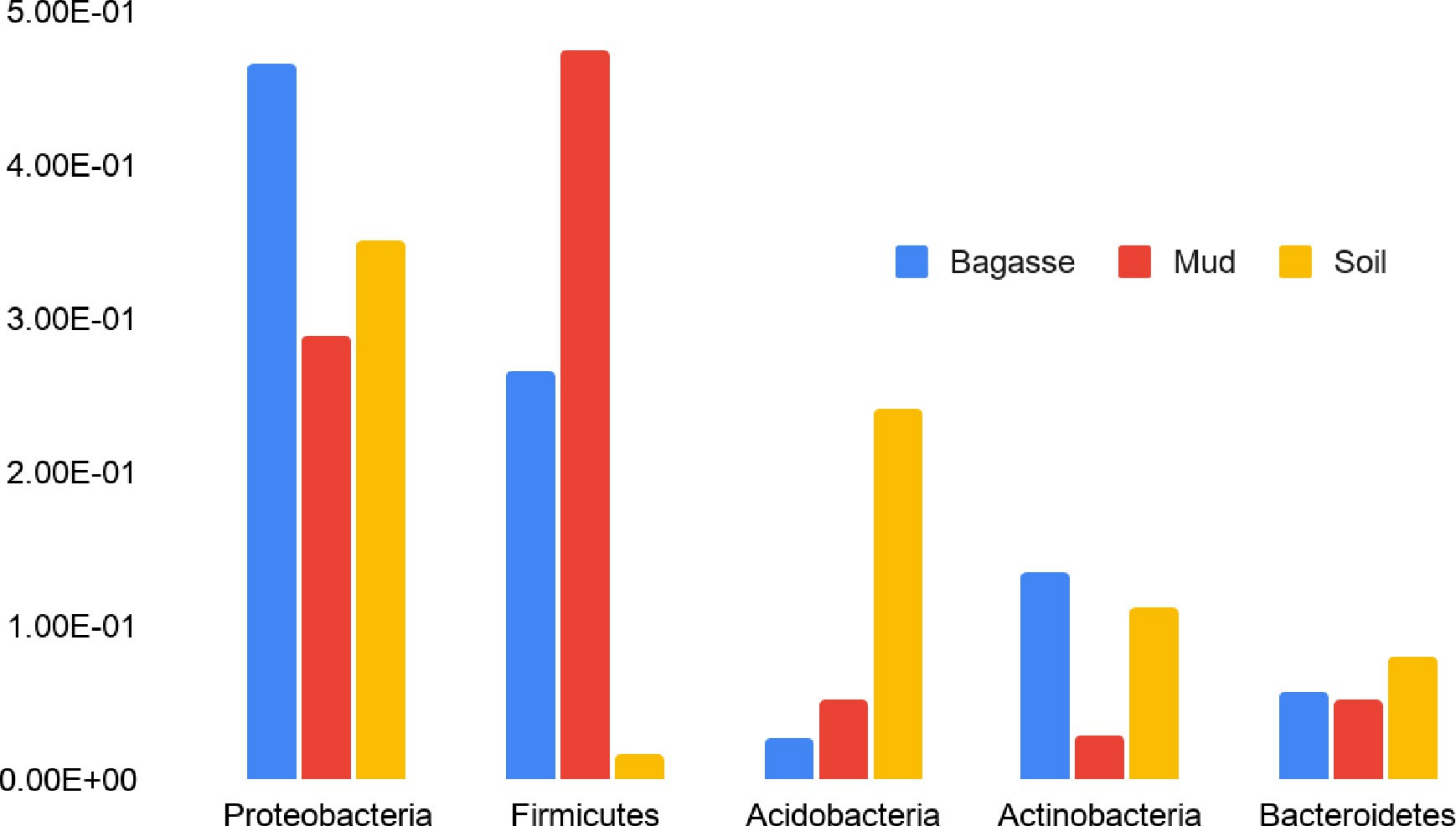
Average relative abundance of 16S rRNA genes from the 5 most abundant phyla.

Overall, the sequencing run yielded approximately 5.5 million usable paired end sequences. The number of reads per sample ranged from 154426 to 520364, averaging 292067. A total of three archaeal phyla and 64 bacterial phyla were detected, with nearly 81% of reads represented by just 5 phyla, each of which representing at least 5% of total reads (Fig 2). 96% of sequence reads were represented by just 11 phyla, each of which represented at least 1% of total reads in all samples. “Cyanobacteria”, which were found to be 5% or more in five samples (2 bagasse and 3 mud), were removed from the microbial analyses because they were found to derive from plant chloroplasts, suggesting incomplete plant (sugarcane feedstock) decomposition in those samples. Several phyla not listed in Fig 2 that deserve further mention are the Chloroflexi which were 5% or more in 3 mud samples, and Chrenarcheota which were 5% or more in 4 soil samples.

The Chrenarcheota, an archaeal phylum known to include chemoautotrophic ammonia oxidizers, accounted for 2.1% of all reads on average, and were mostly found in soil samples. The relative abundance of Chrenarcheota was 4.9% (average; range 3.8–7.1%) of all soil reads, 0.43% of non-soil reads, and 0.11% of mill mud reads. The difference in Chrenacheota reads was highly significant between soil and mud (T test p<0.0001), but not between mill mud and bagasse.

The average relative abundances of the five most abundant phyla are shown in Fig 2 by sample type. When grouped without consideration for aging effects (resulting from environmental exposures, as described in subsequent sections), bagasse was notable for higher relative abundances of Proteobacteria and lower than average levels of Acidobacteria and Actinobacteria. Mill mud had the highest relative abundances of Firmicutes, whereas soils were higher in Acidobacteria than bagasse and mud. The relative abundances of Proteobacteria and Bacteroidetes were not significantly different between sample types (Fig 2).

### Alpha diversity

**Fig 3:**
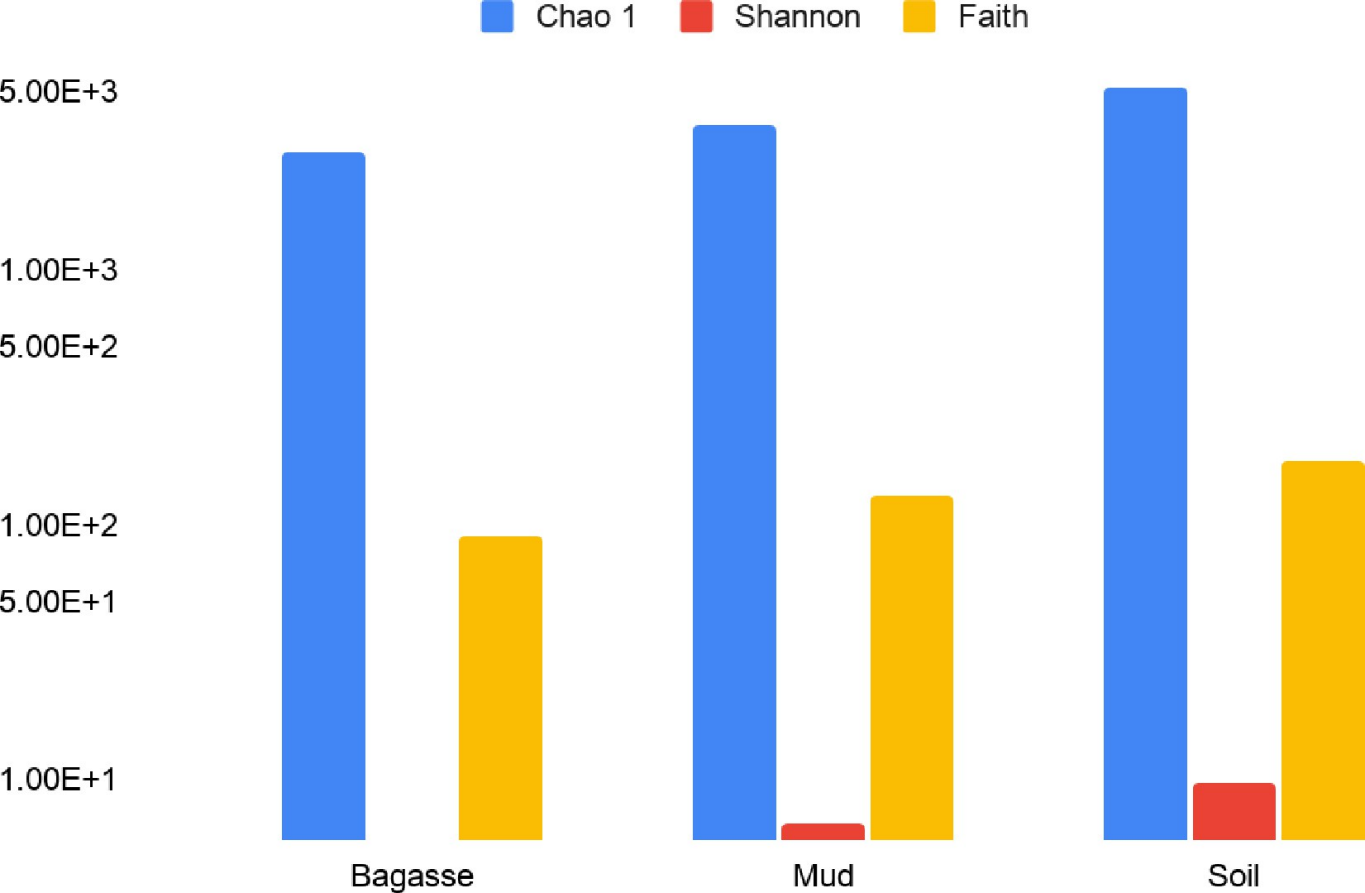
Alpha diversity analyses of samples based on observed 16S rRNA gene (97% similarity).

To gain an ecological perspective on the microbial diversity of samples beyond dominant phyla comparisons, three different alpha diversity analyses were performed on the 16S sequences (Fig 3 and Table 3). Although soils were generally most diverse regardless of the metric, no significant differences were seen between soil and mud using the Chao 1 index (adds weight to rare taxa) or the Shannon index (used to assess evenness and richness). The Chao 1 and Shannon values for bagasse were significantly lower than those for mud and soil (Tukey’s PHT p<0.05). All three sample types were significantly different from each other based on Faith’s index, which focuses on the phylogenetic diversity of the samples. Collectively, mud and soil had similar evenness and richness of a given taxon, including rare taxa. The large Faiths’ values indicate that the genetic distance between the taxa was greater in soil than mud or bagasse.

**Table 3:** Significance of alpha diversity ANOVA and Tukey’s post-hoc test.

	*Chao1*	*Shannon*	*Faiths*
*ANOVA* (p values)	0.0050	0.0075	0.0041
Soil vs Mud	0.1160	0.2708	0.0474
Bagasse vs Soil	0.0006	0.0118	0.0001
Mud vs Bagasse	0.0134	0.0078	0.0285

**Fig 4:**
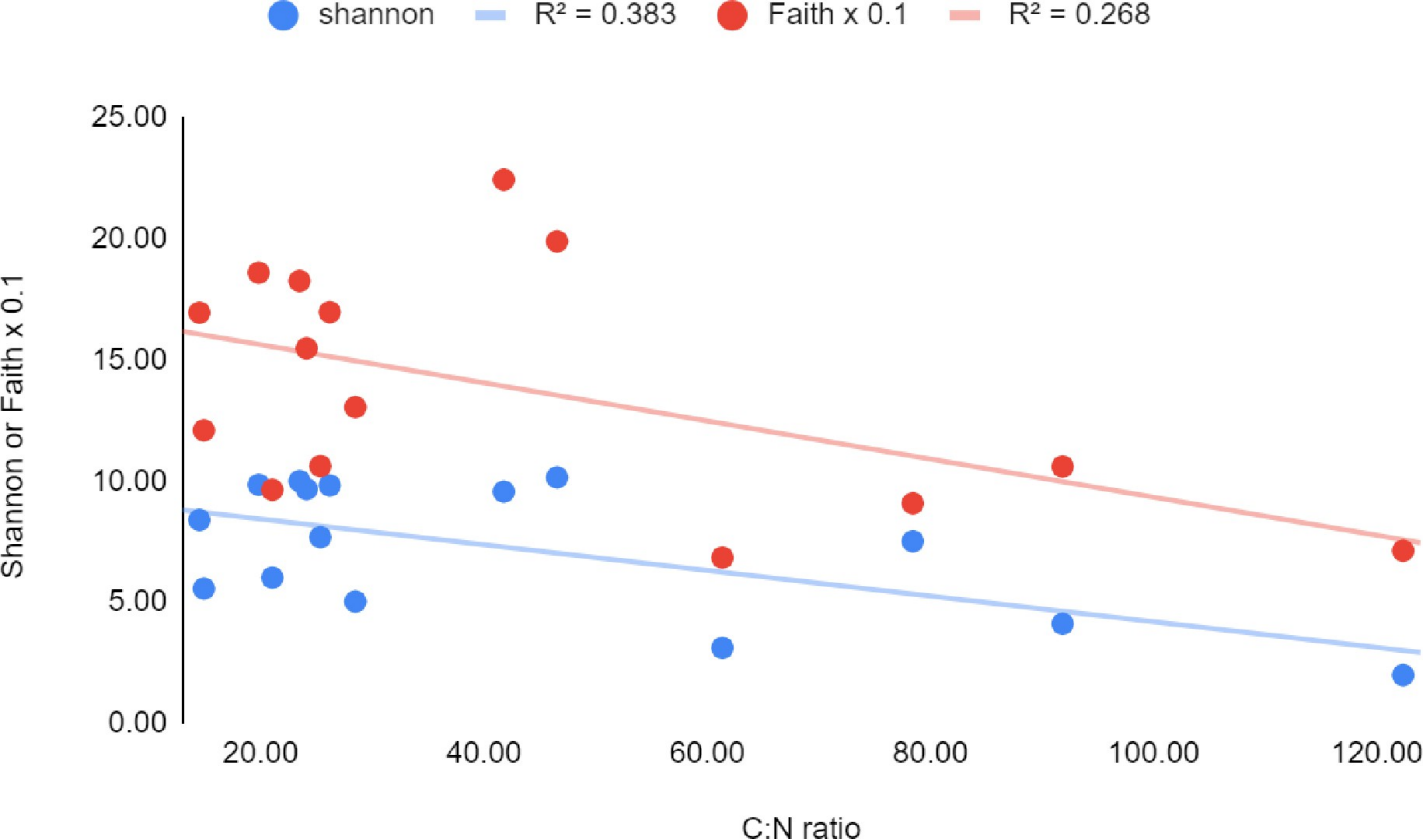
Correlations between C:N ratios and alpha diversity metrics. For ease of comparison, Faith’s index values were divided by 100.

The Pearson Product-Momentum correlation (r) was strongly negative between C:N and both Shannon (-0.99) and Faith (-0.92) indices (Fig 4). No linear correlation was observed, however, between alpha diversity and the chemical parameters representative of soil (pH) or mud (P_2_O_5_). At the phylum level, positive correlations with lower absolute r values were found between pH and Acidobacteria (0.67), Plantomycetes (0.64), and Chrenarcheota (0.63).

### Beta diversity

**Fig 5:**
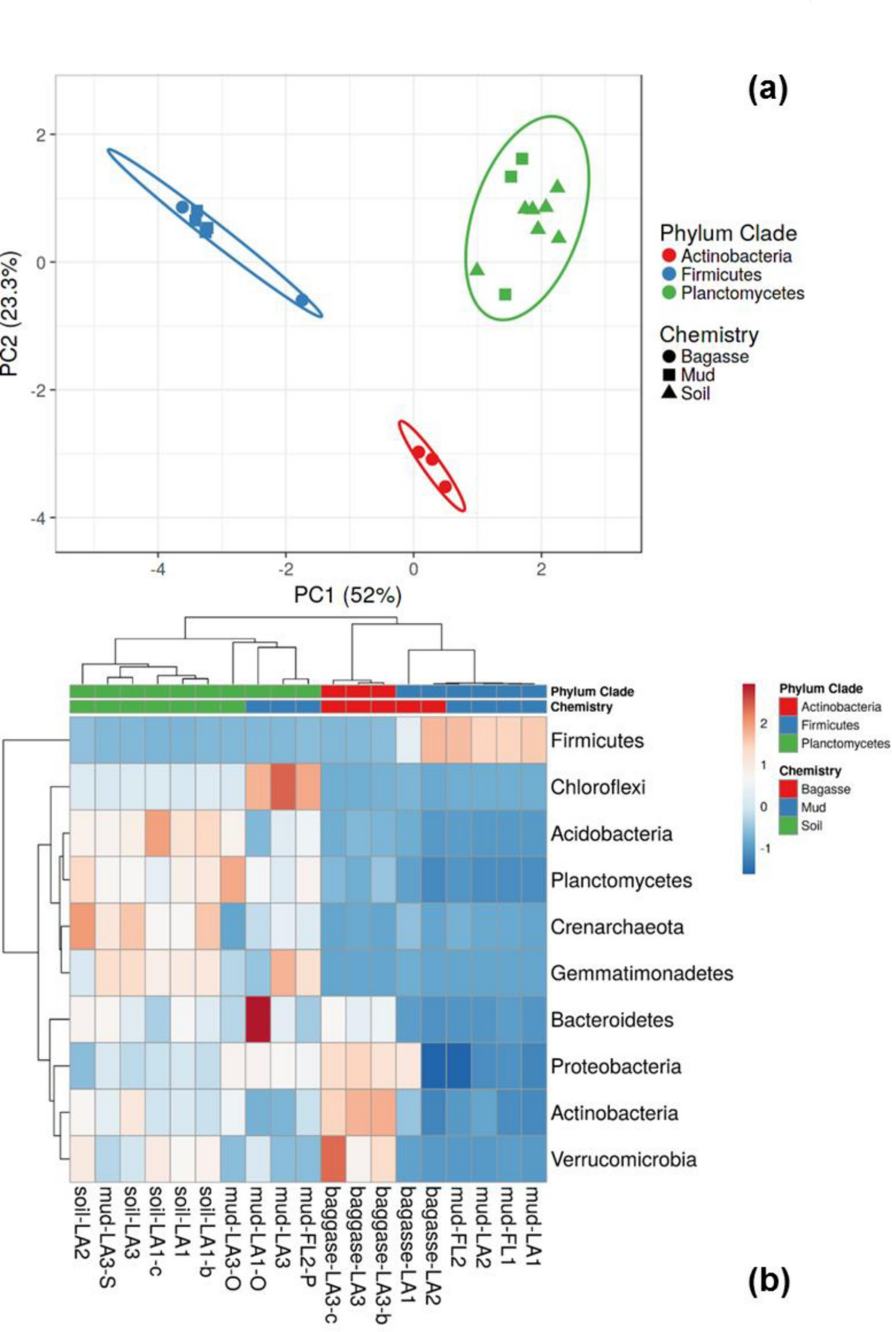
Beta diversity analyses of samples based on the relative abundances of the top 10 phyla. (a) Principal component and (b) heatmap with complete linkage clustering.

Alpha diversity offers the degree (high or low) of diversity as a function of evenness, richness, or phylogeny, without providing a comparison for the community similarity. Beta diversity analyses, in contrast, allow us to compare sample similarity using a PCA plot analogous to the presentation of the chemistry data in Fig 1A. Fig 5A is a PCA plot based on the relative abundance of the 10 most abundant phyla. Three main clusters of samples (colored symbols) are well-separated by ellipses, and represent clades with significantly different relative abundances of Firmicutes, Actinobacter, and Planctomyces. The first component explained 52% of the observed variation, while the second component explained 23%.

Three biological replicates of soil from LA1 in Fig 5B (Soil-LA1, Soil-LA1-b, and Soil-LA1-c) clustered together with all other samples chemically defined as soil (green triangles in Fig 5A) as well as legacy/aged mill mud (mud-LA3-O, mud-LA1-O, mud-FL2-P, and mud-LA3; green squares) that had known prolonged environmental exposure at on-site storage site for solid byproducts from sugarcane processing.

All aged mill mud (italicized samples in Table 1) had a much more diverse community than factory mud (bold text samples in Table 1) as illustrated in the heatmap (Fig 5B). Of all the samples investigated, Mud-LA1-O had the highest level of Bacteroidetes, and contained a more diverse variety of other phyla including Proteobacteria, Plantomycetes, and Chloroflexi than the corresponding factory mud (Mud-LA1). Legacy muds with years of environmental exposure from different locations (Mud-LA1-O and Mud-LA3-O) had similar microbial composition with intermediate levels of Proteobacteria. Those legacy muds only differed modestly from a factory mud aged for 9–10 months outdoor (Mud-LA3), with the latter having higher levels of Chloroflexi and lower levels of Planctomycetes.

In addition to clustering together, aged mud samples (mud-FL2-O, mud-LA1-O, mud-LA3, and mud-LA3-O) all clustered together with the soils, including mud-LA3-S, a mud-amended soil (Fig 5B). Aged muds had similar levels of Planctomycetes to soils, regardless of geographical location. Aged mud samples, however, had higher relative abundances of Proteobacteria and Chloroflexi and less Chrenarcheota than soil samples (Fig 5B). The 16S data demonstrating the similarity of aged mud to soil provided additional insights to changes hinted at by chemical results for aged/legacy muds in Fig 1.

Bagasse collected directly from the factory (bagasse-LA1 and bagasse-LA2) clustered with factory muds regardless of origin (Fig 5A blue squares) and were dominated by Firmicutes. Predominance of Firmicutes in factory mud is consistent with high temperature treatment (≈90°C for 1–2 hr) that would have removed mesophilic bacteria. Three biological replicates of aged bagasse from LA3 (bagasse-LA3, bagasse-LA3b, bagasse-LA3c; Fig 5A red circles) clearly clustered together and were distinct from factory bagasse (bagasse-LA1 and bagasse-LA2, blue squares). The aged bagasse-LA3 also had high relative abundances of Actinobacteria, Proteobacteria, Verucobacteria, and Bacteroides (Fig 5B), but almost no Firmicutes. While bagasse-LA1 (Fig 5A, blue circle) also had high levels of Proteobacteria, it was much more similar to bagasse-LA2, having abundant Firmicutes (Fig 5A, blue circle). Factory bagasse also had lower pH (<5 for bagasse-LA1 and begasse-LA2) than aged bagasse (>6 for bagasse-LA3).

Integrating both chemical and microbial measurements, the distinguishing characteristics for soil and aged mud were Planctomycetes and high pH (~7). Factory muds were notable for high Firmicutes and P_2_O_5_. High C:N was distinctive of all bagasse, but among baggasses, only aged bagasse (bagass-LA3), with its high level of Actinobacteria, had a different microbial signature than factory muds. Collectively, fresh factory mud and factory bagasse (6 far right columns in Fig 5B) shared a similar microbial community associated with the sugarcane factory processes and were dominated by Firmicutes.

## Discussion

To our knowledge, no prior work has systematically investigated the chemical and microbial composition of sugarcane mill mud and bagasse undergoing varying stages of aging after factory production. One related study described the fungal community of composting sugarcane mill mud [9]. In the present study, mill muds collected within the raw sugar production factories were dominated by Firmicutes that survived the extended heat treatment (≈90°C for 1–2 hr) used to produce raw sugar. Similarly, factory bagasse (without aging) was dominated by Firmicutes, despite different chemical characteristics (high C:N for bagasse, high P_2_O_5_ for mud). Although systematic sampling (as a function of time) to follow the trajectory of the same samples as they aged was not logistically feasible for this pilot study, our snapshot of fresh and aged mud and bagasse suggests that, despite their similar initial microbial community, exposure to the outdoor environment leads to distinctive microbial signatures that were likely driven by their different chemical compositions. In fact, the community composition of our aged bagasse (bagasse-LA3) resembled mature compost and older bagasses which are typically dominated by Actinobacteria and Proteobacteria and a decrease in Firmicutes [25–27].

The microbial community of aged food waste composts and bagasse are known to vary over time as well as with depth [18, 26, 28, 29]. Generally, Proteobacteria, Actinobacteria, and Bacteroidetes were the most abundant phyla present in aged food compost and bagasse, though specifics on the age and chemistry of compost and bagasse piles are often not provided [25, 27]. Kanokratana et al. noted that in addition to Proteobacteria, Acidobacteria were present in composting bagasse and that community composition varied with depth and proximity to soil [28]. Rattanachomsrim, however, reported that Firmicutes were abundant in 3-month-old bagasse whose temperature was ~50°C. This suggests that the latter was still in the thermophilic phase of composting [29, 30], which is consistent with the work of Gebbie et al. who recently reported resampling the same undisturbed bagasse pile (~ 8 months apart) at multiple depths [26]. Not surprisingly, they found differences in both bacterial and fungal communities with depth and time. Taken together, the differences in published results from separate studies of single bagasse piles [27–29, 31] are consistent with our observations of bagasse of different ages and from different locations and helps to explain why Firmicutes were largely absent in our aged LA3 bagasse, but present in the LA1 and LA2 bagasse samples that were fresh from the mill.

In conclusion, our combined snapshots of the chemistry and microbiology of samples of different ages suggests that freshly processed mud and bagasse initially shared similar community structures. The mud and bagasse communities then appear to diverge over time, likely due to their chemical [31] and physical differences. In addition to aging and chemical differences, local environmental differences will likely also contribute to community differences between aged mud and bagasse samples from different locations.

While it is difficult to reproduce real world composting conditions in controlled laboratory experiments [32, 33], future efforts to characterize the temporal dynamics of community composition in aging mill muds and bagasse would benefit from paired microbial and chemical/physical characterizations, from the same facility, aged under realistic conditions in sufficient quantities to provide soil amendments for controlled plant growth trials. Far from being a purely academic undertaking, such analyses would help inform practices already commonplace for the management of sugarcane residuals where logistical constraints such as inclement weather, equipment, and/or labor availability may dictate when, how, or if mud and/or bagasse may be field applied. Given that chemical differences alone, were not good predictors of microbial composition of either mud or bagasse, a better understanding is needed of how their microbiomes affect plant yield and soil health.

In conclusion, field samples of soil, bagasse, and mill mud were first grouped based on the chemical composition. Subsequent microbial characterization suggests a similar successional trajectory due to environmental exposure (e.g., aging or soil amendment) even though samples were collected across multiple locations (5 factories) and states (FL and LA) under varying storage conditions. This study offered the following environmental implications to predict the fate of sugarcane mill mud. First, factory-generated microorganisms are unstable and are prone to diversify, regardless of material chemistry. That is, Firmicutes dominated factory samples despite distinctively different chemical compositions of mill mud and bagasse. Second, chemistry drove the distinctive microbial signatures resulting from aging. Nutrient availability in soil is controlled by both chemical and microbial processes [16, 34]. Nutrient availability, in turn, governs the functions of soil microbiome characterized by high diversity and population/functional stability despite active species turnover [14].

Prior reports on soil amendment (fertilizer application) of sugarcane byproducts focused on crop yields and changes in soil chemistry [3, 4, 35–39]. Microbial composition has been characterized, e.g., for compost derived from sugarcane bagasse and other biomass [25, 32, 40–42]. Application rate (how much mill mud is applied on soil) and timing (at which plant growth stage) are expected to influence the efficacy of these applications [43]. One proposed method is to supplement existing fertilizer practice with mill mud amendment to promote crop yield [4]. Concerns have been raised, however, that direct application of mill mud without composting could lead to the growth of harmful fungi [9], though the presence of pathogenic microorganisms is also a concern for composted organic wastes [41]. Regardless of the amendment’s origin, microbes are crucial for releasing the nutrients in soil-applied plant residues, and could play a role in suppressing soil pathogens [41].

## Supporting information

S1 Dataset Underlying chemical and microbial data. (XLSX)
